# Classification of Electronic Health Record–Related Patient Safety Incidents: Development and Validation Study

**DOI:** 10.2196/30470

**Published:** 2021-08-31

**Authors:** Sari Palojoki, Kaija Saranto, Elina Reponen, Noora Skants, Anne Vakkuri, Riikka Vuokko

**Affiliations:** 1 Department of Steering of Healthcare and Social Welfare Ministry of Social Affairs and Health Helsinki Finland; 2 Department of Anesthesiology, Intensive Care and Pain Medicine Peijas Hospital Helsinki University Hospital Vantaa Finland; 3 Faculty of Social Sciences and Business Studies University of Eastern Finland Kuopio Finland

**Keywords:** classification, electronic health records, hospitals, medical informatics, patient safety, risk

## Abstract

**Background:**

It is assumed that the implementation of health information technology introduces new vulnerabilities within a complex sociotechnical health care system, but no international consensus exists on a standardized format for enhancing the collection, analysis, and interpretation of technology-induced errors.

**Objective:**

This study aims to develop a classification for patient safety incident reporting associated with the use of mature electronic health records (EHRs). It also aims to validate the classification by using a data set of incidents during a 6-month period immediately after the implementation of a new EHR system.

**Methods:**

The starting point of the classification development was the Finnish Technology-Induced Error Risk Assessment Scale tool, based on research on commonly recognized error types. A multiprofessional research team used iterative tests on consensus building to develop a classification system. The final classification, with preliminary descriptions of classes, was validated by applying it to analyze EHR-related error incidents (n=428) during the implementation phase of a new EHR system and also to evaluate this classification’s characteristics and applicability for reporting incidents. Interrater agreement was applied.

**Results:**

The number of EHR-related patient safety incidents during the implementation period (n=501) was five-fold when compared with the preimplementation period (n=82). The literature identified new error types that were added to the emerging classification. Error types were adapted iteratively after several test rounds to develop a classification for reporting patient safety incidents in the clinical use of a high-maturity EHR system. Of the 427 classified patient safety incidents, interface problems accounted for 96 (22.5%) incident reports, usability problems for 73 (17.1%), documentation problems for 60 (14.1%), and clinical workflow problems for 33 (7.7%). Altogether, 20.8% (89/427) of reports were related to medication section problems, and downtime problems were rare (n=8). During the classification work, 14.8% (74/501) of reports of the original sample were rejected because of insufficient information, even though the reports were deemed to be related to EHRs. The interrater agreement during the blinded review was 97.7%.

**Conclusions:**

This study presents a new classification for EHR-related patient safety incidents applicable to mature EHRs. The number of EHR-related patient safety incidents during the implementation period may reflect patient safety challenges during the implementation of a new type of high-maturity EHR system. The results indicate that the types of errors previously identified in the literature change with the EHR development cycle.

## Introduction

### Background

The key components of health information technology (HIT) and electronic health records (EHRs) play a crucial role in patient management, care interventions, and effective health care services [1]. The literature indicates that HIT can improve patient safety and quality of care [2-4]. Despite evidence that improvements have helped with the adoption and implementation of EHR systems, EHR adaptation is not without obstacles or challenges [5,6]. EHR adoption may cause unintended consequences, safety risks, and other outcomes [7-9].

Data on error types specifically for high-maturity EHRs [10-12] remain scarce, and available studies have focused on EHRs from the earlier development stages; otherwise, the development stage is not described in detail [13]. Varied patient safety issues related to EHRs and documented in research include poor usability, inadequate communication of laboratory test results, EHR downtime, system-to-system interface incompatibilities, drug overdoses, inaccurate patient identification, care-related timing errors, and incorrect graphical display of test results [14-20].

Many researchers share the view that technology-induced errors arise from several sources in a complex health care environment [6-8,15,21]. Risks associated with EHRs have been identified as being related to technologies, apps, and their use [21-24]. Many EHR errors are latent and involve technological features, user behavior, and regulations, thereby making error anticipation challenging while underscoring the importance of identifying vulnerable areas [25]. The patient safety incident reporting system is fundamental to obtaining and processing patient safety–related information for improving work. Incident reporting aims to detect problems and investigate underlying causes; as a result, there is a possibility of using organizational learning to prevent such incidents from happening again [26-29].

In 2012, the Institute of Medicine recommended that information produced by HIT-related patient safety incidents should be used to improve patient safety [30]. The open sharing of HIT-related patient safety incident data using a uniform structure or other standards could help institutions learn the best practices for EHR implementation. Simultaneously, it is essential to recognize the limitations of patient safety incident reporting to avoid data misinterpretation. However, this information is not shared frequently, so organizations are constantly reinventing the wheel to address EHR issues and improve functionality [2,31]. There is a concern that benefits from HIT-related safety data are lost because of the absence of a mechanism to classify HIT-related events; yet, it is not well established how to define and classify incidents in these systems [19,28,29]. It has been suggested that research evidence, testing, and development of classifications applicable specifically for high-maturity EHRs are needed [10-12,28].

Implementing or upgrading an EHR system is a major endeavor for health care organizations. Decisions on the implementation process, such as user training and customization of the product, can have long-term implications on the usability of EHRs and thus safety related to EHR use [12,32-34]. Our capacity to reap the benefits of new technologies and manage new threats is contingent on understanding the potential threats to patient safety [19]. In the following sections, we describe our study design and results after developing and testing a new problem classification for reporting patient safety incidents while implementing and using a high-maturity EHR system [10-13]. Implementation of this system occurred in a Finnish university hospital with a first go-live phase that began in 2018. For clinical personnel, this meant a change from a previous EHR system to a new high-maturity EHR system. Our research data comprised incident reports from periods as early as 6 months before implementation and as late as 6 months immediately following the beginning of implementation.

### Objectives

The aims of our study are specified as follows:

1. Our primary aim is to develop an error classification applicable to EHR-related patient safety incidents involving high-maturity EHRs.
2. Our secondary aim is to validate technology-induced error classification using real-world patient safety incidents, including the assessment of interrater agreement.

## Methods

### Study Design

A study design was proposed to develop and validate a classification for patient safety incidents. In this study, the concept of technology-induced errors was applied to define EHR-related patient safety incidents [35]. Classifications and taxonomies are used widely in clinical contexts; however, in the literature, they are based on practical needs to standardize medical data in documentation, with less emphasis on theorizing and characterizing classifications and other terminological systems [36,37]. In a clinical setting, classifications can be applied for various reasons, for example, to support clinical thinking to help establish guidelines for diagnosis and treatment [38]. The classification and other core concepts used in this study are listed in Textbox 1. Our primary focus—developing a classification for technology-induced errors—was based on previous research; however, we assumed that further development was required for the classification to be applicable with high-maturity EHRs. At a conceptual level, error classification captures both the instance and its conditions portrayed in patient safety incident reports. However, in the class descriptions, we also used the term *problem* to describe the reporting professional’s experience of a situation that needs to be reported and remedied.

**Key concepts and abbreviations used in this study.**

**Classification (taxonomy)**

- Taxonomies (classifications) are modes of information management that have been used successfully in areas such as medicine and information technology to describe, classify, and organize items based on common features. In this paper, we use the term
  classification
  [36,38,39].

**Technology-induced errors**

- These errors result from the design and development of technology, the implementation and customization of a technology, and the interplay between the operation of a technology and the new work processes that arise from the use of technology [35,40].

**Electronic health records (electronic medical record and electronic patient record)**

- Medical Subject Headings conceptualizes electronic health records as “media that facilitate transportability of pertinent information concerning (a) patient’s illness across varied providers and geographic locations.” Synonyms for electronic health records include electronic patient records, electronic medical records, computerized patient records, and digital medical records. In hospitals, electronic health records are often software apps that contain or interact with other apps. They cover apps for computerized provider order entry, clinical decision support, test results storage, and medication administration systems. These software apps need networked hardware and clinical data structures to operate [41,42]. In this paper, we use the abbreviation electronic health record.

**Electronic health record (Electronic Medical Record) Maturity Model**

- One of the electronic health record maturity models is the Electronic Medical Record Adoption Model, developed by Healthcare Information and Management Systems Society Analytics. It has become a universally recognized maturation model of a hospital’s electronic medical record environment. The Electronic Medical Record Adoption Model is an eight-stage maturation model that reflects hospitals’ electronic medical record capabilities, ranging from a completely paper-based environment (stage 0) to a highly advanced paperless and digital patient record environment (stage 7) [10-12]

Our starting point for the classification development in this study was based on previous research by Sittig and Singh [21,22]. The initial coding framework followed the structure of the Finnish Technology-Induced Error Risk Assessment tool comprising eight main categories: EHR downtime; system-to-system interface errors; open, incomplete, or missing orders; incorrect identification; time measurement errors; incorrect item selected; failure to heed a computer-generated alert; and failure to find or use the most recent patient data [14,15,43]. This tool-based coding framework was refined and extended through analysis and development by our research team based on the clinical experience of medical doctors using the studied EHR.

In addition to data-based analysis, to review and update the classification based on the latest research, articles on EHR error types were gathered from PubMed (MEDLINE complete). We searched for EHR error types with Medical Subject Headings using the keywords *electronic health records*, *patient safety*, and *medical informatics*, and *technology-induced error* was applied as a search term, although it is not yet a Medical Subject Heading term.

### Study Materials and Research Context

We collected patient safety incident reports, which illustrate typical errors with an older EHR system and a new system to be implemented in a Finnish university hospital. The hospital district is among the largest in Finland, with 25,916 employees. In 2019, 680,000 patients were treated at the hospital, with 2.9 million outpatient visits and 92,000 surgeries performed. Since 2007, the hospital has been using a fully paperless EHR system [15,44]. The implementation of a new high-maturity EHR (Healthcare Information and Management Systems Society 6-7) started in 2018 at the first site to cover emergency services and several medical specialties. Data on all types of patient safety incident reports and 12 medical specialties were retrieved on July 17, 2020, from the university hospital’s database.

The research data used comprised real-world patient safety incident data to develop and assess the emerging classification identified in the literature and in previous studies and expanded in our research. The Finnish patient safety incident reporting model and instrument, called HaiPro (Awanic), was developed in 2006. It is anonymous, nonpunitive, and not integrated into any EHR system. All personnel—including all nurses, physicians, and academic hospital workers (eg, pharmacists)—have been trained and are encouraged to report patient safety incidents through HaiPro. Although HaiPro contains structured data, the main content of incident reports is descriptive [44].

The research review process of the university hospital organization approved the study protocol (study permission update March 23, 2020, License org.id/200/2020). In the collected research data, no connection to patients or professionals exists because of the nature of the anonymized data, which do not contain any identification details. Psychiatric reports were excluded because of data sensitivity. To allow for comparisons in terms of the number of patient safety incident reports, we included all safety incidents reported through the HaiPro system during the 6-month period before the implementation of a new EHR system in 2018. A similar selection process with a full reading of reports was also applied during the implementation.

### Data Cleaning, Data Analysis, and Validation

**Figure 1:**
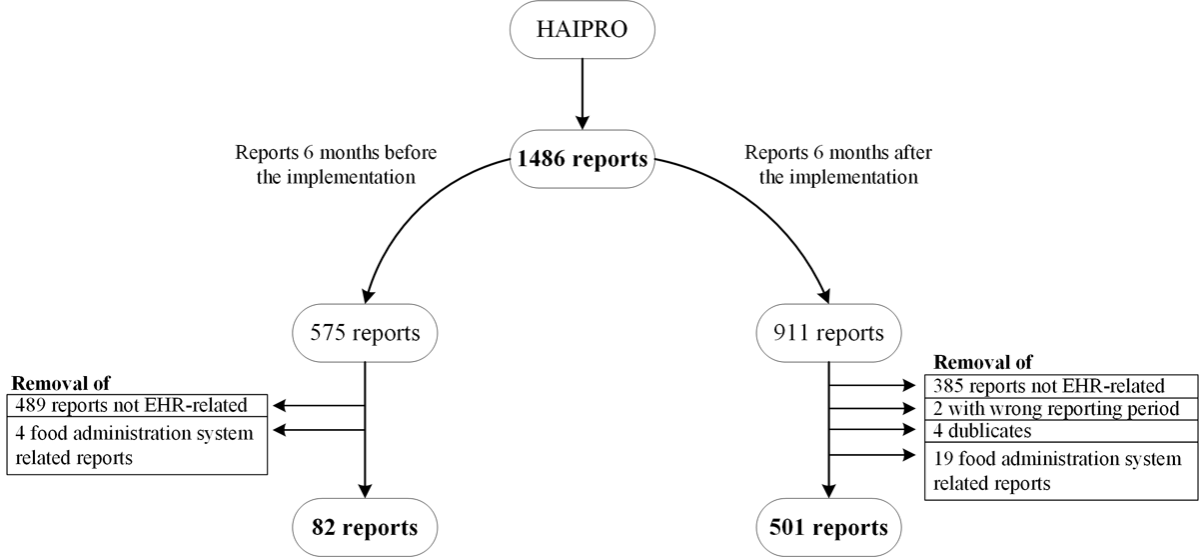
The process of categorizing the reports for data analysis with 82 reports from before the implementation and 501 reports from after the implementation remaining for a blinded review with the proposed classification. EHR: electronic health record.

The incident report data were processed before starting the analysis, as shown in Figure 1. To clean up the research data, patient safety and informatics experts read all the reports in the database thoroughly to identify the EHR-related cases. Duplicates and reports concerning food administration information systems have been removed. All reports that met the inclusion criteria (EHR related) were selected for this study. Two clinical experts (medical doctors) with 2 years of experience in implementing and studying EHR systems and extensive experience with patient safety reporting made detailed and documented decisions on cases in which the definition of EHR-related error incidents was not clear. Our research team comprised 3 clinicians with 3 clinical informatics and classification experts.

For our research purposes, 82 reports from before the implementation and 501 reports from after the implementation remained for a blinded review with the proposed classification. The process of classifying the data and reviewing the results are presented in Textbox 2. A more detailed process of data analysis and validation is provided in Multimedia Appendix 1. During the data analysis and review of the research team, we developed the original classification by adding several classes or subcategories. Finally, we validated the classes based on the distribution of incidents.

**Study design for data analysis and validation.**

**Patient safety incident report data quality analysis and validation**

- Agreement upon preparatory classes and their descriptions; common rules for classifying data
- Blinded reviews of the data with the classification; research team agreements for classification revisions and refinement
- Blinded testing of revised classification
- Classification validation and results from data analysis finalized

First, to perform a classification-based analysis, the research team agreed on preparatory classes and their descriptions at the start of data analysis, as well as common classification rules. During the next research phase, 2 researchers with substantial experience in classification development and informatics independently reviewed a set of reports and applied the classification in a blinded fashion, along with 2 researchers with clinical experience. Disagreements were discussed among the research team, and the study design was adjusted accordingly. After each set of test rounds, the interpretation of the classes was discussed to update the wording of the classes and their descriptions. Altogether, seven classification rounds for multidisciplinary consensus and validation procedures were conducted to perform the iterative development of the emerging classification (Multimedia Appendix 2). Selecting the same main category created a match while choosing a different category or failing to find the category at all was viewed as a nonmatch. Disagreements were discussed by the research team. Percentage agreement was applied to perform the interrater reliability measurement.

During the third research phase, informatics and clinical experts tested the revised version of the classification to validate the results. Finally, the data analysis was completed after a 7-month research period that ended in March 2021. The research team reviewed the final results and revised the classification by refining the descriptions of the final classes.

## Results

### Overview

Here, we present the results from the patient safety incident report data analysis based on the results from the error classification that emerged during our iterative data analysis. In addition to presenting the results from validation, we also present observations regarding the development of the classification. Development needs for an original structure were realized during the analysis, and more subclasses were needed.

### Data Analysis

The total number of all types of patient safety incident reports (excluding psychiatry) during a 1-year period was 1486. There were 38.69% (575/1486) reports during the 6-month period before the implementation of a new EHR system, of which 14.2% (82/575) of cases were related to EHRs. Altogether, 61.31% (911/1486) of reports were entered into the database 6 months after the implementation of a new EHR system, of which EHR-related incidents totaled 54.9% (501/911).

The total reporting volume during the implementation phase increased by 58.5%, with the number of cases related to the EHR system during the postimplementation period was five times higher (510%) than before implementation.

During classification, 14.8% (74/501) of EHR-related incident reports were rejected and thus remained unclassified. Decisions concerned situations wherein information was insufficient to classify the event reliably, or it was possible that the notification was not related to the EHR system.

The interrater agreement was 97.7%. During the blinded review, 10 discrepancies between reviewers were found in the final data (n=427), which were accepted for the classified data. Moreover, the previously mentioned rejected incident reports created discrepancies during the classification.

### Validation of Classification

Our final analyses of EHR-related error types comprised 427 classified incidents. A detailed distribution (classification and frequencies of error types by main categories and subcategories) is provided in Multimedia Appendix 2.

The downtime (8/427, 1.9%) category was associated with the problem of logging into a single part of the EHR system or application (2), or the entire EHR system (3), whereas the presence of planned downtime existed only in one report. An unplanned downtime did not exist in the research data. During classification with our data, we noticed that not all incidents fit the existing subcategories. We added a new subcategory for data entry during and after a period of downtime, and we split the system-logging-problem subcategory to relate to all or part of the system in use to better capture issues with a high-maturity EHR system.

Among the 22.5% (96/427) of interface problems, 36% (35/96) of incidents were found in the category of data transfer between different EHRs within the same organization. This was caused partially by the implementation that occurred in the first hospital site at that time, and multiple EHR systems were still in use in the entire hospital district. Data transfer within the different components of the same patient information system accounted for 39 incidents. On the basis of our data and classification reviews, we added several subcategories to capture the complex interface issues in EHR adoption, in which transference occurred as a change from one EHR to another in a highly competent environment of clinical and HIT ecosystems.

Problems with timing functions accounted for 5.9% (25/427) of cases. Most of the reports (n=21) concerned changes in medication and treatment scheduling because of the programming logic in the EHR. This category’s original classes worked well with the data, but we decided to update the class descriptions to better reflect high-maturity EHRs.

The largest number of cases, at 20.8% (89/427) of reports, was related to the medication section, whereas the smallest number (1/427, 0.2%) was found in the mixed patient record problems category. We noticed that the original classification did not cover these incidents adequately to capture the complex issues; thus, a new class was added after reviewing this incident type in our research team.

The usability problem category (73/427, 17.1%) covered notifications as follows: most reports concerned problems with missing, incomplete, or wrong alarms, or alarm fatigue (n=29) and problems finding data (n=30). Problems with decision support accounted for two reports and printing problems in 11 cases. One of the usability problems remains unspecified. For the subcategories related to alarms, we updated the class descriptions and clarified the characteristics of decision support–related issues as they relate to other alarms or system notifications. After discussing the data analysis findings within the research team, we separated workflow problems from the usability class. As a problem category, workflow problems are typically more complex than mere usability issues.

Clinical workflow problems using EHRs were the underlying causes of errors in 7.7% (33/427) of cases, and competence problems were identified in 5.4% (23/427) cases. These were divided into two subcategories, of which 16 reports cited a lack of education. Obstacles to competence development caused by EHRs were cited in seven incidents. Within the emerging classification, workflow problems were deemed complex situations in which EHRs played a clearly identified role. Typically, these cases occur when the system cannot support the clinical workflow, or when the workflow is interrupted.

The documentation category (60/427, 14.1%) comprises four subcategories, the largest of which turned out to be unspecified documentation issues in its 27 cases. The lack of data structure, errors in data structure, or interpretation problems with data structure appeared in 20 notifications, whereas clinical classification deficiencies were found in one report. The loss of recorded information during documentation was identified as the cause of incidents in 11 cases, a well-established category in previous research. On the basis of our data analysis, we decided to clarify the class descriptions to make it easier for reporting professionals to differentiate documentation incidents from usability problems. Simultaneously, subcategories were added to capture the manifold issues of documenting. Unrecognized problems with data loss form a separate main class, comprising 2.6% (11/427) of reports. Class descriptions for data loss are also updated to indicate clear differences in usability problems.

An examination of the data revealed that 1.9% (8/427) of cases were related to the category of general situations, in which patient safety is threatened because of the introduction of a patient information system. This class can be used to capture incidents that seem to portray situations involving the poor organization of work during ongoing implementation phases in complex health care environments that, based on our data, typically may include demanding activities such as multitasking, problem solving, and clinical reasoning.

The classification and frequency of error types in the main and subcategories are provided in Multimedia Appendix 2. After the research team agreed to classification updates, the classification system comprised 13 main classes, with additional subcategories for several classes.

## Discussion

### Principal Findings

There is a need to integrate research into the design, development, and implementation of health technologies for improving their safety and reducing technology-induced errors [35]. The evolution of knowledge in this area has witnessed growth [35], but a classification suitable for EHR users’ clinical practices is needed to derive maximum benefit from safety information reported through these means [19,28,44]. During this study, error types were adapted iteratively after several test rounds to develop a classification for reporting patient safety incidents in the clinical use of high-maturity EHRs. Some of the categories for error types have been identified in the scientific literature [13-20]; thus, their rationale exists. However, reliable classification work requires a solid knowledge of the features of an EHR system. In this study, an effective understanding of the content of problem reports was ensured by a multidisciplinary research team that included 3 physicians using the EHR system daily.

As the classification work progressed, one compromising agreement had to be made to continue classification development and validation with these particular data. According to the data, the medication section of the studied EHR system caused incidents for which it was not possible to detect a specific root cause. However, it was clear from the descriptions that the incidents were caused by features in the EHR system’s medication section. As a result, a category was created for these incidents, but a deeper analysis in future research is needed to address the underlying problems with the medication section. Only some incidents related to the medication section were related to a lack of competence and classified accordingly. Finally, the manner in which the study was conducted was time consuming in terms of manual classification and review by the research team, but such a methodological approach was very profitable in practice. However, it is evident that the new emerging classification requires further validation in different health care contexts and with different high-maturity EHR products. Moreover, clinical users should test the classification so that its functionality and applicability can be assessed from the clinician’s perspective in real patient care situations.

The number of EHR-related patient safety incidents during the implementation period was five-fold as compared with the preimplementation period, which can be viewed only as an indicative figure with respect to the actual situation. However, while analyzing possible reasons for increases in safety events, how members of a clinical team are organized and assigned, and how patient care is coordinated and delivered, is of paramount importance [32]. In this study, because of illustrative incident descriptions, a category, *general situation of endangering patient safety due to the introduction of an electronic health record*, was developed. On the basis of professionals’ descriptions, the implementation of a new EHR system may disrupt the conventional ways of organizing and coordinating patient care; thus, it is justified to include the category to examine the wider implications of the implementation of the EHR system from the perspective of corrective actions [27,44].

Of the 427 classified patient safety incidents, usability problems accounted for 73 (17.1%) incidents, documentation problems for 60 (14.1%) incidents, medication section for 89 (20.8%) incidents, and clinical workflow problems for 33 (7.7%) incidents. Downtime problems were rare (8/427, 1.9%), and unlike in previous studies [15,43], unplanned downtime did not exist. Owing to decreases in unplanned situations, we assumed that the hospital competence for EHR implementation has developed with experience from previous implementations. However, despite the new EHR system being a high-maturity EHR system, further efforts are recommended to improve its usability, make the medication section more user friendly, and devote more attention to the needs and perspectives related to clinical workflow in the development of EHR systems [12,13,16-18,20,23,32]. In doing so, the EHR system provides even more benefits as a tool for clinicians to improve patient safety [22].

### Limitations

The study had several limitations: causal attributions for HIT-related risks and safety incidents are difficult to identify, as they generally involve interactions between technical and nontechnical factors, which are notoriously difficult to separate [22]. The development of the classification was time consuming, and practical challenges were encountered in the application of the classification. The biggest obstacles arose from the readymade data, which included the professionals’ own descriptions of the incident. Not all professionals described the incident’s features in sufficient detail. Typically, this caused a situation in which the research team could not always definitively ascertain which category applies to an incident. To ensure the reliability of the results, 74 incidents were rejected when the research team members held detailed discussions after the blinded review. Therefore, it is important to ensure that the organization continues to pay attention to making sufficiently detailed descriptions to benefit from the reporting [27,40,44].

Moreover, it should be noted that the nature and well-known limitations of patient safety incident reporting should be considered while interpreting the volume of incident data. Reports do not provide exact frequencies of incidents; consequently, data do not provide exact error rates, but rather a descriptive analysis of typical EHR-related safety problem types [26-28,44].

### Conclusions

The broad spectrum of patient safety incidents is best understood by assessing data from multiple sources using a uniform classification, and this study proposes such a system for high-maturity EHR systems, which are known contributors to patient harm. However, this study’s results indicate that the error types previously identified in the literature change and are specified with the development cycles of EHR maturity. Technology-induced errors in high-maturity EHRs include at least suboptimally developed workflows, usability design challenges, and interface and documentation problems. Unlike previous studies, there were no unplanned downtimes. Further research is recommended to evaluate the suitability of the classification for clinical use and its possible wider applicability in health care systems.

## Appendix

Multimedia Appendix 1 Classification and data analysis process for the iterative development of electronic health record patient safety error classification.

Multimedia Appendix 2 Classification of error types for electronic health record–related incidents by main and subcategories, which are identified with class numbering, for example, for the first class, the main category is “1” and the subcategories are “1.1” and “1.2.” Table columns illustrate class identifiers, names, and respective class descriptions. The table also provides the number (N) of classified error reports per class category.

## Abbreviations

EHR: electronic health record
HIT: health information technology
