# Supplementary figures and images for: Classification of Electronic Health Record–Related Patient Safety Incidents: Development and Validation Study
Source: JMIR Med Inform. 2021 Aug 31;9(8):e30470. doi: 10.2196/30470 (PMC8441612; doi:10.2196/30470)

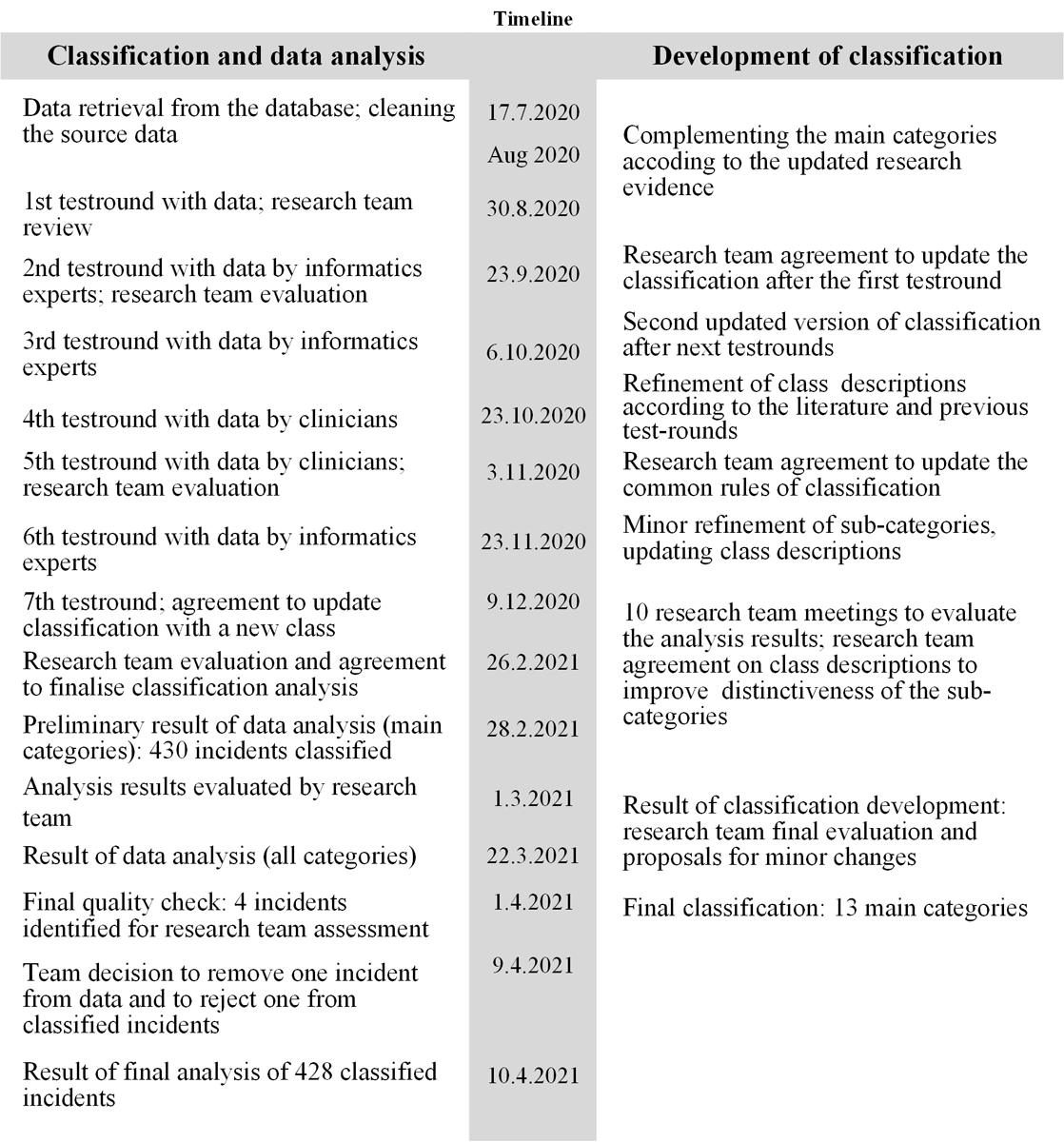

Supplement: Multimedia Appendix 1 [file medinform_v9i8e30470_app1.png]
