# Supplementary material for: Classification of Electronic Health Record–Related Patient Safety Incidents: Development and Validation Study
Source: JMIR Med Inform. 2021 Aug 31;9(8):e30470. doi: 10.2196/30470 (PMC8441612; doi:10.2196/30470)
Supplement: Multimedia Appendix 2 [file medinform_v9i8e30470_app2.docx]

| Class ID and name | Class description | N |
| --- | --- | --- |
|  |  |  |
| **1 Downtime problems** | downtime or login problems that prevent retrieving, viewing, processing, or entering information | 1 |
| 1.1 Unplanned downtime for the patient information system, lasting several hours | a problem that prevents retrieving, viewing, processing, or entering information into an EHR for several hours | 0 |
| 1.2 Less than an hour of unplanned downtime for the patient information system | a problem that prevents retrieving, viewing, processing, or entering information from or into an EHR for less than an hour | 1 |
| 1.3 Prevented from logging into the entire system due to technical reasons related to the information system | a problem limited to logging into a single part of the EHR system or application  (Note: This is not actually system downtime.) | 2 |
| 1.4 Inability to log into part of the system due to technical reasons related to the information system | a problem limited to the area of use (module) or view of the individual system; usually affects many users simultaneously, e.g., the login problem is limited to a wake-up call  (Note: This class does not cover downtime issues.) | 3 |
| 1.5 Planned downtime for the information system | a problem that prevents information from being retrieved, viewed, processed, or entered during a planned downtime; the problem manifests itself in the deficiency of contingency functions observed during downtime, e.g., during a planned outage, there is no operating model in accordance with the contingency plan, or the key information needed for care is not available in print or in the backup information system | 0 |
| 1.6 Data entry after downtime | a problem concerning situations in which patient data are recorded in the EHR at the end of a downtime period, resulting in deficiencies or errors in key patient data, e.g., the data are recorded on paper and stored in the EHR after the downtime; data generated in medical devices are not stored in the EHR during downtime, in which case, some data are lost | 1 |
| **2 Interface problems** | problems concerning situations in which data retrieval is delayed, the data are (unpredictably/unintentionally)modified, or data transmission is blocked completely | 1 |
| 2.1 Problem with data transfer between different organizations | a problem concerning situations in which no functional interfaces exist between organizations, or no interface exists at all | 15 |
| 2.2 Problem with data transfer between different patient information systems in the same organization | a problem concerning situations in which no functional interfaces exist, or no interface exists between EHRs from the same organization | 35 |
| 2.3 Problem with data transfer within different parts or components (modules), or with viewing patient information | a problem concerning situations in which no functional interfaces exist between different parts or components of the same EHR; the medical device-EHR interface also can be affected, e.g., the cause may involve system implementation regarding data transfer between departments or views of professional groups. | 39 |
| 2.4 Problem with data transfer related to national information services or a regional information system | a problem concerning situations in which no functional interfaces exist between the EHR and the centralized service (e.g., Finland’s national, centralized Kanta eHealth information services or the regional information system Navitas), causing a delay in retrieving data from the centralized information systems, manifested in EHR slowness  (Note: This category does not include situations in which Kanta or Navitas was not used originally. | 6 |
| **3 Problems with timing function** | problems that cause situations in which the EHR generates errors due to the timing function, typically related to medication, laboratory tests, and imaging procedures | 1 |
| 3.1 Scheduling change programming issue | a problem that causes system-based situations in which the EHR schedules events independently regardless of the information entered, manifested in incorrect dosing or procedure times, extra doses, or duplicate interventions, or the system programs events to continue even if they are due to end, e.g., medications or examinations are scheduled differently than what was programmed in the prescription data. | 21 |
| 3.2 Other timing-change issue | a problem that causes a system-based situation in which an incorrect timestamp appears for an unidentifiable reason in the EHR logging, e.g.,  the timestamp of patient record entries for diagnostic information is stored incorrectly for an unidentified reason.  (Note: The class usually does not appear as a stand-alone, but rather as a secondary category. Workflow problems [Class No. 6] may involve a timing problem as one component, but if so, the incident is reported primarily as a workflow problem, and a timing problem is reported as a secondary category). | 3 |
| **4 Medication section problems** | problems that cause situations in the medication section of the EHR in which prescription and record information is not stored as intended, or apparent changes have been made due to an unidentified system-related reason  (Note: This is often a problem that hinders management of overall medication. The class does not necessarily appear as a stand-alone, but may be complemented by a secondary class. | 89 |
| **5 Usability issues** | problems that concern the EHR’s usability, i.e., situations in which the EHR is not easy to use or complicates or prevents achievement of the goal set for use of the EHR, or the system does not support job execution expectedly | 1 |
| 5.1 Problems and deficiencies related to alarms | problems that concern situations in which the user does not receive the required alarm, or the alarm is false or illogical; the category also includes alarm fatigue, in which the user skips a necessary alarm because several alarms are used, or they have been deemed unnecessary previously, e.g., the attitude that "all alarms are useless" and that relevant alarms do not stand out | 29 |
| 5.2 Problem with decision support | a problem that concerns situations in which the user does not receive reminders or instructions that should be included in the functionality of decision support, e.g., drug interaction functionality does not work the way it should.  (Note: This category does not include situations in which the decision support does not include the latest data.) | 2 |
| 5.3 Problem with finding data | a problem that concerns situations in which information appears difficult for the user to perceive; information is difficult to find or must be "dug out,” e.g., by knowing a random path for finding it within the system display; or information is displayed illogically and is difficult to use (e.g., the user must "click" several windows open), drop-down menus are too narrow, abbreviations are used in drop-down menus, the font layout is too tight, or the placing of specific data items varies in different views, e.g., different windowing of data intended for different professional groups causes a situation in which information is displayed in different ways, and some information may be lost or ignored when there is no place for it in a particular view | 30 |
| 5.4 Printing problem | a problem related to situations in which, when printing, the information changes or is difficult to find on the printout when the information is printed in a conflicting or incorrect format, e.g., a paper printout is printed for patient-transfer purposes, resulting in several unnumbered pages with information that lacks structure.  (Note: Problems with printing medicine labels fall into this category.) | 11 |
| **6 Clinical workflow problems** | problems that concern situations in which the clinical work procedure has not been implemented in the EHR to match the clinical workflow, i.e., the system does not support job execution, and work continuity is disrupted, e.g., a fundamental process step (such as a patient transfer situation) needs to be performed, but is not possible or the EHR does not support it. Examples of Class No. 6 can be related, e.g., to the medication section when discontinuing overlapping medication during intensive care is inconsistent with home medication. The prescription already is registered for the patient’s home care, and the system does not allow for flexibility, but requires the drug to be removed from prescriptions. Due to the lack of a logical workflow, the system attracts the use of ways which are not aligned with guidelines when the EHR functionalities for the next work step do not open due to a deviation from the previous work step.  (Note: The situation may include individual features from other categories, such as a timing problem (Class No. 3) or usability (Class No. 5). | 33 |
| **7** **Documentation problems** | problems that concern situations involving a system error, obstacle, or ambiguity in entering data; this problem is noticed when the recording cannot be completed as intended | 4 |
| 7.1 Lack, error, or interpretation problem in data structure | a problem that concerns situations in which no field to enter data exists in the EHR, and as a result, the information is entered in the wrong place, e.g., in a free text note or in a comment field, in which the next user cannot find the information. This problem also can concern situations in which the data structure does not meet the need, is incorrect, or is subject to interpretation, in which case, it is not clear which place of entry should be chosen  (Note: An incident is not classified in this category if the user purposely records deviating from the commonly agreed-upon policy.) | 20 |
| 7.2 Classification deficiency | a problem that concerns situations in which a classification deficiency is found in the EHR, e.g., the ICD-10 code or procedure code is missing or incorrect, in which case, the data cannot be recorded correctly | 1 |
| 7.3 Loss of recorded information during documentation | a problem that concerns a documentation situation in which the professional notices a loss of information when saving or, e.g., moving to the next section | 11 |
| 7.4 Other unspecified documentation issues | problems that applies to other situations in which documenting cannot be performed correctly or completed for a system-specific reason that the user does not recognize | 24 |
| **8 Unrecognized problems with data loss** | problems that concern situations in which information already stored in previous work steps is found to be lost in the EHR; this data loss is not detected until later, e.g., when the professional performing the next work step does not receive the information he or she needs, such as an order or a referral | 11 |
| **9 Problems with mixed patient records** | problems that concern a situation in which data from two different patients’ records are combined or mixed for system-based reasons, e.g., information about a patient can be found in another patient's report  (Note: This error is rare in Finland. The system-specific patient identifier [identification number] used in other countries is often the root cause of this problem. | 1 |
| **10 Problems with competence or training** | problems that concern situations related to training and skills | 23 |
| 10.1 Problem with competence in using EHRs | a problem related to the individual user's own or organization-oriented activities in which the error can be deduced from a lack of training or expertise  (Note: The category also includes problems due to incompetence by a system support person. This category does not include usability issues.) | 16 |
| 10.2 Obstacle to competence development caused by EHRs | a problem related to situations in which the system features make it difficult to apply the training received so that the activity can be carried out in accordance with the instructions, or the training received does not correspond to the current version of the system, and the user feels that the information system cannot be used in accordance with the given training, and development of competence does not solve the problem | 7 |
| **11 General situations endangering patient safety due to implementation of an electronic health record** | problems that concern situations in which it is not possible to determine reliably which part of the error is due to the EHR, the user or underlying factors, or the overall system in the health care unit, especially in the implementation of the EHR, which can endanger a patient | 8 |
| **12 Other unidentified error conditions** | problems that concern situations in which no applicable category exists, but the incident is recorded, and this category is proposed for further development of the classification | 0 |
| **13 Rejected notifications** | problems that concern situations in which not enough information is available to classify the EHR-related incident reliably. | 74 |
